# Marine Macroalgal Polysaccharides in Nanomedicine: Blue Biotechnology Contributions in Advanced Therapeutics

**DOI:** 10.3390/molecules31010175

**Published:** 2026-01-02

**Authors:** Renu Geetha Bai, Surya Sudheer, Amal D. Premarathna, Rando Tuvikene

**Affiliations:** 1 School of Natural Sciences and Health, Tallinn University, Narva mnt 29, 10120 Tallinn, Estoniarantuv@tlu.ee (R.T.); 2 Chair of Biosystems Engineering, Institute of Forestry and Engineering, Estonian University of Life Sciences, Kreutzwaldi 56/1, 51006 Tartu, Estonia

**Keywords:** nanoparticle, biocompatible, antioxidant, anticancer, antiviral, antibacterial

## Abstract

Marine macroalgae represent a versatile and sustainable platform within blue biotechnology, offering structurally diverse polysaccharides that are making significant contributions to next-generation therapeutical applications. Algae are rich sources of high-value biomolecules, including polysaccharides, vitamins, minerals, proteins, antioxidants, pigments and fibers. Algal biomolecules are widely explored in modern pharmaceuticals due to their range of physiochemical and biological properties. Recently, algal polysaccharides have gained increasing attention in nanomedicine due to their biocompatibility, biodegradability and tunable bioactivity. The nanomedical applications of algal polysaccharides pertain to their anti-coagulant, antiviral, anti-inflammatory, antimicrobial and anti-cancer properties. In this review, we discuss some major macroalgal polysaccharides, such as agar, agarose, funoran, porphyran, carrageenan, alginate and fucoidan, as well as their structure, uses, and applications in nanomedical systems. Both sulfated and non-sulfated polysaccharides demonstrate significant therapeutic properties when engineered into their nanotherapeutic forms. Previous studies show antimicrobial potential of 80–90% antiviral activity > 70%, significant anticoagulant activity, and excellent anticancer responses (up to 80% reductions in cancer cell viability have been reported in nanoformulated versions of polysaccharides). This review discusses structure–function relationships, bioactivities, nanomaterial synthesis and nanomedical applications (e.g., drug delivery, tissue engineering, biosensing, bioimaging, and nanotheranostics). Overall, this review reflects the potential of algal polysaccharides as building blocks in sustainable biomedical engineering in the future healthcare industry.

## 1. Introduction

Macroalgae, commonly known as seaweeds, are one of the major sustainable marine resources with significant potential as a rich source of chemically diverse, bioactive, high-value products. The presence of numerous nutrients such as dietary fibers, vitamins, minerals, proteins, peptides, lipids, fatty acids, polyphenols and antioxidants has promoted their use, mainly in Asian countries. The lower occurrence of chronic diseases, such as cancer, cardiovascular diseases, metabolic syndrome, diabetes, osteoporosis, etc., in Asians has been associated with a healthier diet profile including seaweed consumption [1,2,3,4]. In addition to food and feed, algae are used in adhesives, medicine, cosmetics, paper making, biofuels, and biofertilizers. In comparison to land-plant-based foods, seaweed-based foods contain several macrominerals (Na, K, Ca, Mg) as well as essential and trace elements (Fe, Zn, Mn, Cu) [5]. The scientific reports on the human digestibility, bioactivity and bio accessibility of algal biomolecules support the use of algae as food in a healthy diet [6]. The nutraceutical and therapeutic potential of seaweeds correspond to the presence of micro/macro minerals, polysaccharides (agars, alginates, carrageenans, fucoidans, xylans, ulvans, laminarins), dietary fibers, proteins (phycobiliproteins), amino acids, lipids and fatty acids, vitamins, polyphenols and pigments [7].

Pharmaceutical industry has a growing interest in natural polysaccharides due to their excellent bioactivities in therapeutic fields as antitumor agents, anti-diabetes agents, anticoagulant agents, and vaccines, as well as synergistic comprehensive treatments along with currently available drugs [8]. Unlike terrestrial polysaccharides, seaweed polysaccharides, both sulfated (carrageenan, fucoidan, funoran related galactants/porphyran, ulvan) and non-sulfated (alginate, agar, agarose), exhibit direct structure–function relationships and corresponding biological interactions, which are relevant in therapeutic applications. Recent reviews on seaweed-derived polysaccharides prove their potential in advanced therapeutic formulations though functionalization or combination [9,10]. The anti-cancer potential of algal polysaccharides is utilized along with chemotherapy to reduce its toxic effects, by improving intestinal flora and immunity [11,12,13]. Algal polymers have attracted a lot of attention in wound healing, drug delivery and tissue engineering due to their antibacterial, antiviral, anticancer, anti-inflammatory, anticoagulation, immunomodulation, and cell proliferative effects, along with biocompatibility, biodegradability and drug-carrying capacity [12,14,15,16,17].

Advances in nanotechnology have expanded their relevance, enabling the design of nanocarriers, hydrogels, scaffolds and composite systems for drug delivery, tissue engineering, regenerative medicine and theranostics. Rapid expansion of this advanced nanotherapeutic platform of algal polysaccharides is due to the properties of the algal polymers, such as predictable gelation, mild processing conditions, high water affinity and wider biocompatibility. A number of applications such as hydrogels, wound dressings, cell encapsulations and injectable matrices have been developed utilizing these properties [18,19,20,21]. Apart from providing structural support (scaffold or dressings), algal polysaccharides provide a synergy-enabling matrix for loaded drugs, antimicrobials, and immunomodulators, thus promoting the targeted delivery of therapeutics supporting nanomedicine strategies.

A structured analysis of peer reviewed literature about macroalgal polysaccharides and their bioactivities and nanomedical applications was utilized to prepare this review. Publications from 2000 were considered to include both foundational and recent advances. About 13 articles are older than 2000. Literature searches were conducted using Web of Science, Scopus, PubMed and Google Scholar. The keywords used include macroalgal polysaccharides, seaweed polysaccharides, alginate, agar, agarose, carrageenan, fucoidan, porphyran, ulvan, nanomaterials, nanoparticle, nanomedicine, drug delivery, wound healing, tissue engineering, antimicrobial, anticancer and nanotheranostics. The article selection followed a PRISMA-inspired process including the identification of records, duplicate removal, screening of titles and abstracts and full text assessments. Studies are included based on their relevance, clarity, polysaccharide structure details, bioactivity, nano synthesis, or therapeutic potential. Non-peer-reviewed sources and articles lacking critical methodological information were excluded. A greater emphasis was placed on the publications from 2020 onwards, to reflect the current trends and translational considerations.

This review systematically explained the transformative potential of macroalgal/seaweed polysaccharides in next-generation nanomedicine. The novelty of this review lies in the integrated focus on: (1) structure–activity relationships of clinically relevant polysaccharides (carrageenan, agar, fucoidan alginate, porphyran etc.) and their in vitro and in vivo bioactivities; (2) eco-friendly synthesis of metallic (Ag and Au) and magnetic (Fe_3_O_4_) nanoparticles using macroalgal polysaccharides as biotemplates; (3) advanced therapeutic platforms including antimicrobial nanodressings, osteoconductive scaffolds and pH-responsive drug delivery systems; (4) molecular mechanisms causing tissue-regeneration and immunomodulatory effects; and (5) translational challenges with emphasis on scalability, reproducibility and regulatory considerations. By bridging marine biotechnology and nanotechnology, this review highlights macroalgal polysaccharides as sustainable solutions to current limitations in drug delivery, regenerative medicine, nanomedicine, and antimicrobial resistance, while addressing the global demand for environmentally friendly healthcare technologies.

## 2. Algal Biotechnology and Algal Polysaccharides

Algal biotechnology involves the utilization of algae (both microalgae and macroalgae) or their derivatives to create or modify various products. Algal phylogenetic diversity depends on their habitat, morphological, physiological and biochemical properties. Algal biotechnology deals with developing new methods in culturing algae, harvesting, processing, identifying the properties at molecular level, exploring applications of algae, and developing new products [22,23]. Algae constitute a group of more than 40,000 species, which includes unicellular organisms such as diatoms to seaweeds extending over meters. They vary depending on pigments, habitats (freshwater, saltwater, extreme environments), and the taxonomic group they belong to. Macroalgae are mostly classified as green (Chlorophyta), red (Rhodophyta) and brown algae (Phaeophyta) based on their pigments. Unlike plants, algae do not have leaves, stems or roots; however, they carry a vital role in marine productivity. The large forms of algae, which are extensively scattered in the ocean, either into considerable depths, free-floating or attached, are referred to as seaweeds which are among the main biomass producers [24]. Considering the sustainable nature of algal biomaterials, review articles on the production, extraction and characterization strategies of algal polysaccharides, nutraceutical applications, food industry, bioplastic production, medical applications are gaining wider attention [25,26,27,28,29]. In this review, we focused on nanomedical applications, whereby we critically evaluate algae-based nanomaterial synthesis and the therapeutic effect of various nanoforms of algal polysaccharides.

### 2.1. Agarans (Agar, Agarose, Funoran and Porphyran)

#### 2.1.1. Agar and Agarose

Agar is a gelatinous hydrocolloid composed of sulfated galactans, synthesized by many red algal species as their major cell wall matrix component. Agar is extensively used as a gelling, stabilizing and thickening agent, where the marketable value of this polysaccharide evaluated by their agarose (non-sulfated fraction) content and gel quality. Owing to its exceptional rheological properties, agar is utilized commercially for applications in food, cosmetic, pharmaceutical, biomedical and biotechnology industries. In the global market scenario, the agar-based industry mainly focuses on seaweed materials with greater agar yield and improved gelling properties [30]. The first source of agar manufacturing was *Gelidium* species and later *Gracilaria* became the main seaweed for making food-grade agar. *Gracilaria*, *Gracilariopsis*, and *Polycavernosa* are the major agar-producing algae [31]. Red algae sources including *Ahnfeltia plicata*, *Gracilaria caudate*, *Gracilariopsis chorda*, *Gracilaria gracilis*, *Gelidium vagum*, *Gracilaria tenuistipitata* are also used for agar production [32,33,34,35,36,37].

**Figure 1:**
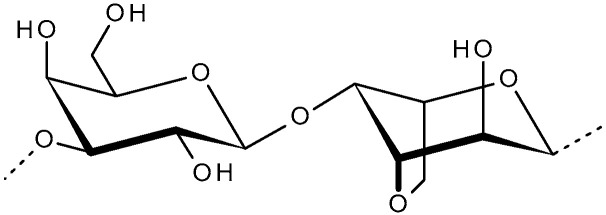
Disaccharide repeating unit structure of agarose.

The chemical structure of agar is characterized by the repeating units of d-galactose and 3,6-anhydrous-l-galactose with a few deviations, and by a low ester sulphate content. Agarose is the purified form of agar with a linear structure made of agarobiose (Figure 1). These polysaccharides can be sulphated to variable degrees. In the past three centuries up to the 1990s, world agar production was monopolized by Japan [38]. Agar, as a hydrophilic colloid, is insoluble in cold water but soluble in boiling water. Considering its concentration and gelation properties, a 1.5% agar solution is transparent but, when cooled to 34–43 °C, it forms a firm gel. The ash content of agar is usually between 2.5 and 4%, but a maximum 5% ash content is acceptable for agar [39]. The biosynthesis of agar in seaweeds is highly influenced by genetic alterations, developmental stages and environmental conditions. Similarly, depending on the extraction techniques utilized, the quality and yield of the agar vary. Moreover, other factors, such as the physiological states of seaweed, abiotic and biotic conditions, climate influences, etc., regulate the yield and quality of agars [40].

#### 2.1.2. Funoran

Funoran is a polysaccharide extracted from the genus *Gloiopeltis* spp. of the Endocladiaceae family. This red algae family includes a group of edible seaweed species extensively used in industries such as food, cosmetics and medical products. Funoran is a sulphated polysaccharide and chemically heterogeneous in structure. The main chain consists of major repeating units of (1→3)-linked β- and (1→4)-linked α-galactose (or 3,6-anhydride). These polysaccharides have antibacterial, anti-inflammatory and anticoagulant potential. Based on molecular weight changes, the biological properties of funoran were found to be varied [41,42,43].

The structural characterization and composition analysis of funoran fractions were evaluated after the cold and hot extraction from *Gloiopeltis* species (*G. furcata*, *G. tenax* and *G. complanata*). The chemically heterogeneous funorans showed very high polydispersities. From the various characterization analyses, the basic units of the *Gloiopeltis* polysaccharides are found to be 3,6-anhydro-α-L-galactose and β-D-galactose-6-sulfate. The biological activities of *Gloiopeltis* polysaccharides were analyzed and funoran exhibited potential antibacterial, anti-inflammatory, anticoagulant, hypocholesterolemic and antioxidant activity [44]. Funorans from the various *Gloiopeltis* species were investigated to analyze the rheology and thermal properties, and the gelation property of funoran was found to be a slow process that is significantly dependent on the existence of cationic substances [45].

The in vivo investigation of funoran on Ehrlich ascites carcinoma and solid Ehrlich, Meth-A fibrosarcoma, and Sarcoma-180 tumors showed significant inhibition of cancer cell growth. In mice models, funoran expressively induced the development of delayed-type hypersensitivity reactions to sheep red blood cells. Intraperitoneal administration of funoran improved the spleen weight of mice and the transformation from lymphocytes to plasma cells in the spleen. This investigation supported the antitumor effect of funoran according to its impact on the augmentation of T-helper, T-cytotoxic, and NK cells [46].

#### 2.1.3. Phorphyran

Porphyran is a sulphated polysaccharide from the Porphyra seaweed species of the order Bangiales. It is the major constituent of the famous Japanese dried algal food ‘Nori’. In addition to the dietary fibers, the presence of protein, polysaccharides, vitamins and minerals makes these algae of nutraceutical importance in the food, cosmetic, medical and industrial fields [47]. Porphyra is among the algae with the highest total polysaccharide content [48]. The chemical structure of porphyran is like that of agarose, composed of a linear backbone of alternating 3-linked β-D-galactose and 4-linked 3,6-anhydro-α-L-galactose units. The l residues include α-L-galactosyl 6-sulfate units, and 3,6-anhydrogalactosyl units [49]. Porphyran has proved various pharmaceutical properties such as antiallergic, antioxidant, antitumor, anticancer, antifatigue, antiulcer, antibacterial, anticoagulant, antiviral, antihyperlipidemic, macrophage stimulation (immunostimulant), hypotensive, and hepatoprotective activity [47,50].

Porphyran is extracted from different types of *Porphyra* such as *P. umbilicalis*, *P. haitanensis*, *P. suborbiculata*, *P. tenera*, *P. yezoensis*, and *P. vietnamensis*. However, the main sources of porphyrin are *P. yezoensis* and *P. haitanensis*. The degradation of porphyrin results in oligo-saccharides called oligo-porphyran (OP) with strong high biological activities due to the decrease in molecular weight and increase in the degree of sulfation [48,51].

The in vitro antitumor activity is investigated using porphyran from *P. yezoensis* Chonsoo2 on the cancer cell lines Hep3B, HeLa and MDA-MB-231 and normal human liver cell HL-7702, where an inhibitory effect was observed in cancer cell growth—significantly, towards Hela cells, whereas the normal cells were not affected [52].

In vitro evaluation of the wound healing activity of porphyran was tested using an IEC-6 cell line (intestinal epithelial cells-6), which displayed enhanced cell migration, protein expression, proliferation and improved epithelial healing [53]. In vivo investigation on Wistar albino rats and albino mice models supported the improved immunomodulatory activity of porphyrin [54]. *Porphyra yezoensis* polysaccharide exhibited excellent repair activity towards the oxalate induced oxidative cellular damage occurred in renal epithelial cells. When tested on human kidney proximal tubular epithelial (HK-2) cells, porphyrin showed a molecular-weight-dependent repair activity towards the cells [55]. In another study by the same team, the nano-calcium oxalate monohydrate (COM)-crystal-induced toxicity was also repaired with the use of degraded porphyran [56].

### 2.2. Carrageenans

**Figure 2:**
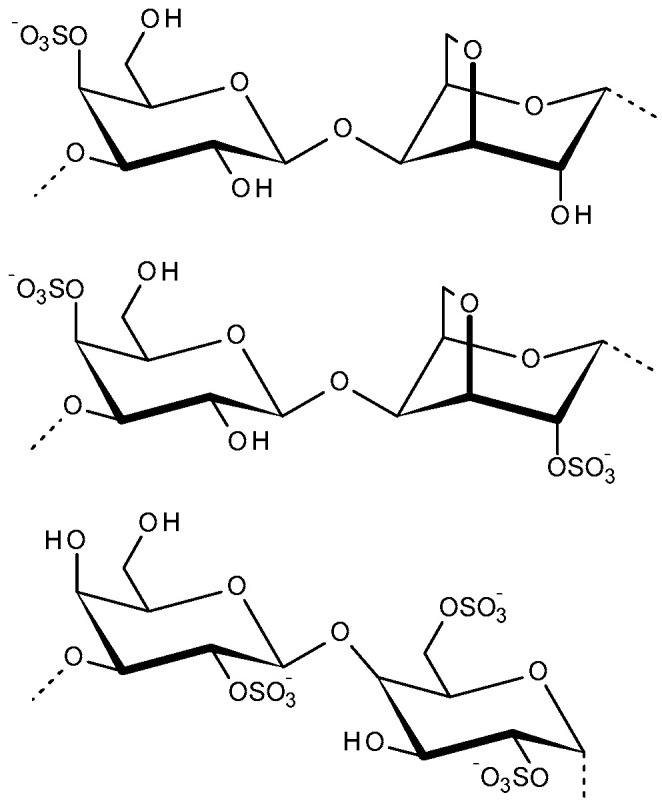
Disaccharide repeating units of kappa, iota and lambda carrageenans (top structure—kappa, middle structure—iota and bottom structure—lambda).

Carrageenans are natural sulfated polysaccharides, extracted from specific red seaweeds, with ester sulfate groups, and 3,6-anhydrogalactose. Carrageenans are water-soluble, linear, sulfated polygalactans. The ester sulfate content of carrageenans is 15–40% and the average relative molecular mass is above 100 kDa. Carrageenans’ structure is comprised of alternate copolymers of α-(1→3)-d-galactose and β-(1→4)-3,6-anhydro-d-galactose. The main types of carrageenan are kappa, iota, and lambda (κ, ι, and λ), and they vary in the number and position of the ester sulfate groups on the repeating galactose units (Figure 2). The κ-carrageenan has only one negative charge per disaccharide, it produces strong, rigid, brittle thermo-reversible gels, and it has outstanding film-forming properties. The ι-carrageenan produces soft elastic thermo-reversible gels, whereas λ-carrageenan is nongelling. Carrageenans are extracted from various red algae such as *Furcellaria lumbricalis*, *Coccotylus truncatus*, *Kappaphycus alvarezii*, *Chondrus armatus*, *Hypnea musciformis*, and *Tichocarpus crinitus* [57,58,59,60,61].

Carrageenans are used in the food industry as thickeners, stabilizers, and emulsifiers. Since carrageenan can strongly interact with proteins, structure and stability are improved when introduced into processed foods. It is also used in food products including pie fillings, chocolate, milk beverages, salad dressing, jelly and processed meat. Similarly, it is used in toothpaste, cosmetics, lubricants, and air freshener gels. Carrageenan-based gels are also used in biomedical applications as a carrier of microbes or to immobilize cells [62,63]. Depending on many factors, the composition and properties of carrageenan preparations vary—depending, for example, on the growth conditions of seaweed, the extraction process, and treatment. Industry-purpose-based preparations are often blended varieties of carrageenans or in combination with other hydrocolloid products. The dispersibility, solubility, solution viscosity, gel strength, hydration rate, and protein interactions of carrageenans could be regulated via blending or processing steps. Food-grade potassium salts and sugar are usually added to standardize the carrageenan products to adjust their viscosity or gel characteristics. Carrageenan products are water soluble and quite stable in a range of pH values; due to the ionized sulfate half-ester groups they are negatively charged, which facilitates the linear configuration [63,64,65].

The use of carrageenans in animal feed proved safe for oral administration without any adverse effects on the gastrointestinal tract or immune system [66]. Carrageenans also possess antitumour and antiangiogenic features, which are evaluated using in vitro and in vivo analysis [67]. The immunopotentiation and immunosuppressive actions of carrageenans have also been investigated, showing the potential of carrageenans in stimulating the immune system [62]. Carrageenans use in the cosmetic industry is based on their physical and functional abilities and antioxidant activity. Carrageenan is utilized in antiaging and anticarcinogenic activity. Due to their gelling nature, carrageenans help in attaining perfect texture and consistency in cosmetic products and they are also used in skin lotions, shaving foams, toothpaste binders, etc. Hybrid carrageenan in the food industry is known as weak kappa or kappa-2 carrageenan [62].

### 2.3. Alginate

**Figure 3:**
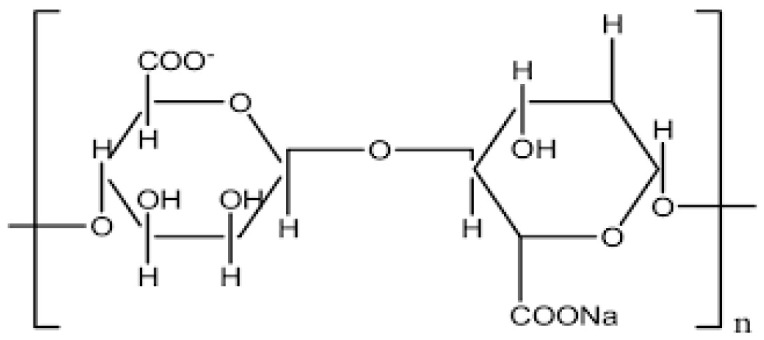
Disaccharide repeating unit of sodium alginate’s structure.

In 1881, E.C.C. Stanford, a British chemist, described the most abundant polysaccharide—alginate—in brown algae, comprising up to 40% of the dry matter. This polysaccharide is a structural component of the cell walls and is located in the intercellular matrix in the form of salts of alginic acid as a gel containing sodium, calcium, magnesium, strontium and barium ions. The term alginate is used for the salts of alginic acid, all derivatives of alginic acid, and alginic acid itself. Alginates are linear polysaccharides, composed of β-D-mannuronic acid (M) and α-L-guluronic acid (G) residues linked via 1→4 glycosidic bonds. The residues are arranged in a block structure of a homopolymer or heteropolymer. Consequently, the alginate backbone consists of sequences of blocks of M-blocks corresponding to mannuronic acid or G-blocks corresponding to guluronic acid, and sections of alternating sequences (e.g., MG, MMG, GGM) [68,69,70] (Figure 3).

Depending on the block structure and molecular size, the viscosity and gelation vary. The GG block binds divalent cations such as calcium ions strongly; thus, due to the metal chelation property, they form strong yet brittle alginate gels akin to an egg-box gel microstructure, where the M-block or MG-block is involved in weak and relatively elastic gel formation [71]. The properties of alginates are influenced by factors such as the ratio of M-G blocks, the sequence of monomers, length of blocks, molecular weight and the source of alginate. Owing to its gelling, viscosifying and stabilizing properties and ability to retain water, alginate is extensively used for industrial applications. The main commercial form of alginate is sodium alginate, although other forms include alginic acid and its calcium, ammonium, and potassium salts, or esters of alginic acid, such as the propylene glycol alginate [72]. Alginate is also used for the encapsulation of cells and enzymes for various advanced biomedical applications. The viscosity of alginate usually depends on the polymer length; however, the fraction and distribution of G-residues determine the gel-forming and water-binding properties and the degree of immunogenicity [73].

Alginates are generally extracted from species such as *Laminaria* spp., *Macrocystis* spp., *Ascophyllum*, *Eclonia*, *Lessonia*, *Durvillea*, and *Sargassum*. Even though the main source of alginates is brown seaweed, alginates are also produced using a biosynthesis approach by bacteria such as *Pseudomonas aeruginosa* and *Azotobacter vinelandii* [74,75]. However, the molecular weight of alginates obtained from bacteria possesses a high degree of polymerization and high molar masses of algae, which are different from that of algae. Moreover, bacterial alginate lacks the oligomeric G sequences in its structure, and they are used commercially for various industrial applications [76]. Alginate is mainly used in food and pharmaceutical industries such as gelling agents, film, stabilizers, viscosifiers, and therapeutic agents. Enzyme-degraded alginate oligomers are an example of enhanced bioactive products [77]. The extensive applications of alginates are due to its compatibility with a range of materials including preservatives, sugar, oils, fats, synthetic resins, latices, waxes, thickeners, pigments, surfactants, alkali metal solutions, plasticizers etc. [78]. Alginate solutions usually show stable properties at room temperature in a pH range of 5.5 to 10. The most stable form of alginate is sodium alginate, whereas the least stable is alginic acid. Regarding the industrial applications, 50% of alginate products are used in the textile industry, 30% in the food industry, and the remaining 20% of alginates are used in the medical, cosmetic, and pharmaceutical industries [79].

### 2.4. Fucoidan

Fucoidan is a sulfated polysaccharide extracted from marine brown algae, sea urchins and sea cucumber. In brown algae, fucoidans usually constitute up to 25–30% of the algal dry weight. As an edible algal component, fucoidan is a part of east Asians’ food culture. The characteristic properties of fucoidan extend its applications in the food industry, nutraceutical, pharmaceutical industry, and cosmeceuticals [80,81,82,83].

**Figure 4:**
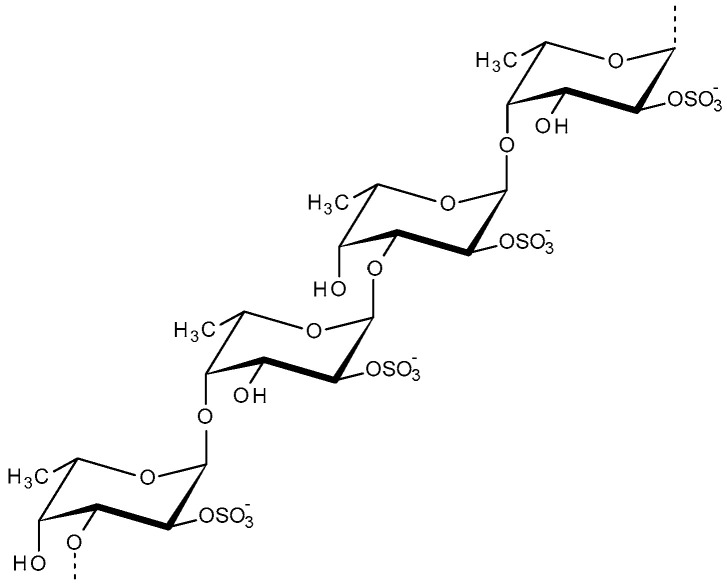
Structure of fucoidan.

The term “Fucoidan” exists alongside fucan, sulfated fucan, and fucosan. The fucoidan structure is mainly composed of L-fucose and is a complex, irregular and rather heterogeneous polysaccharide. This monosaccharide could be linked to galactose, rhamnose, or both galactose and rhamnose (rhamnoglactofucan). It may also contain some acetyl groups. The two main backbones of fucoidan are (1→3)-linked-L-fucose residues or alternating (1→3)- and (1→4)-linked-L-fucose residues (Figure 4). The structural variances of the main chains of fucoidans are due to different biosynthetic pathways. The pharmacological potential of this polymer relies on its antimicrobial, anti-inflammatory, antiviral, anti-thrombotic and anti-cancer properties. Fucoidan is also used as a biocompatible smart carrier for drug delivery purposes. Moreover, active targeting of fucoidans towards cancer cells via P-selectin receptors over-expressed on cancer cells is another promising factor, potentially widening its applications [82,84,85].

When brown seaweeds such as *Ascophyllum nodosum*, *Fucus vesiculosus*, *Cladosiphon okamuranus*, *Sargassum kjellmanianum*, and *Sargassum thunbergii* were used to extract fucoidan, L-fucose was found in a range of 12.6 to 36.0%, and the sulfate content ranged from 8 to 25%. The biological properties of fucoidan depend on the degree of sulfation, structure, molecular weight and extraction process. Numerous biological activities, including antitumor, immunomodulatory, antiviral, antithrombotic, anticoagulant, antithrombotic, antioxidant and antilipidemic effects, were observed in fucoidan, probably due to the presence of sulfate groups. Specifically, in cancer therapy, fucoidan inhibits cancer cell proliferation through a series of cascades such as cell cycle arrest and induced apoptosis, modifying growth-signaling molecules and preventing metastasis and angiogenesis. Compared with traditional anticancer drugs, fucoidans show promise for anticancer therapy by targeting several cellular signaling pathways regulating growth factors, tumor cell survival factors, protein kinases, receptors and transcription factors [86,87,88,89].

Algal-derived polysaccharides can be classified as those lacking intrinsic antimicrobial activity and those with antimicrobial activity, corresponding to their chemical structure and functional group composition. In recent studies, sulfated and sulfonated polysaccharides such as carrageenan, fucoidan and ulvan displayed antimicrobial potential due to polyanionic sulfate groups supporting electrostatic interactions with positively charged microbial surfaces and causing cell disruption. Antimicrobial activity has been shown to be related to the degree of sulfation, sulfation pattern, molecular weight and charge density. Non-sulfated algal polysaccharides such as alginate and laminarin exhibit minimal antimicrobial action if not chemically modified. This structure–bioactivity relationship further supports synergy when another antimicrobial component is included in the advanced therapeutic preparations [90,91,92].

## 3. Nanotechnology and Algae: Biosynthesis of Nanomaterials

Nanotechnology is an expanding field of science that deals with particles of sizes 1 to 100 nm. Apart from their bulk counterpart, nanosized materials possess specific physical, chemical and biological properties, which enable them to be potential candidates in advanced medical applications [93,94,95,96,97]. Depending on the physical parameters such as the concentration, metal precursors, reducing agent, temperature, pH, and time, the size, shape and stability of the metal will vary [98]. Synthesis of nanoparticles can be achieved in three main ways, using physical, chemical and biological methods. The biosynthesis of nanoparticles is a popular field, which is supported by green chemistry. Numerous studies report the biosynthesis of gold, silver, and carbon nanoparticles and their industrial and biological applications [99,100,101].

Owing to their high capacity for metal accumulation, algae enable the green synthesis of metallic nanoparticles; this is known as phyconanotechnology. Algae-based biosynthesis has advantages such as the accessibility of bulk culturing, good energy efficiency, low-temperature processes, fewer toxicity-related concerns, and eco-friendly processes and products. The formation of nanoparticles by algae includes two methods—intracellular and extracellular biosynthesis. The key steps in this process are: (1) the preparation of the algal extract in an aqueous or organic solvent at the optimized temperature, pH and duration; (2) the preparation of molar solutions of metal precursors under stirring or without stirring for the specific duration under controlled parameters. Moreover, based on the algal type, nanoparticles could be formed either in an extracellular or an intracellular mode. During extracellular formation, the bioactive phytochemical components in the algal extract (reducing sugars, polysaccharides, peptides, proteins, pigments) interfere with the metal precursor and form nanoparticles. However, in the case of intracellular metallic nanoparticle formation, algal metabolism and pH were found to play major roles in the reduction of metal ions. i.e., photosynthesis and respiration might be responsible for the reduction of metallic ions [102,103].

Nanoparticles of gold and silver, inert metals with antimicrobial and cancer therapeutic potential, are extensively applied to identify their medical applications. Marine algae *Gracilaria corticate*-based biosynthesis was used to develop gold nanoparticles (AuNPs), which could have antimicrobial and antioxidant applications [104]. Similarly, an aqueous extract of the brown algae *Cystoseira baccata* was utilized for AuNP biosynthesis and its anticancer activity was assessed in colon cells. The anticancer study showed strong cytotoxicity against Caco-2 and HT-29 cell lines and no toxicity in the healthy cells. Moreover, the developed AuNPs induced apoptosis activation via the extrinsic and mitochondrial pathways [105].

A variety of algae such as *Acanthophora spicifera*, *Chlorella pyrenoidusa*, *Kappaphycus alvarezii*, *Laminaria japonica*, *Stoechospermum marginatum*, *Sargassum myriocystum*, *Sargassum wightii*, *Sargassum tenerrimum*, and *Turbinaria conoides* and blue green algae including *Microcoleus chthonoplastes*, *Phormidium valderianum*, *Plectonema boryanum*, and *Spirulina platensis* were used for the biosynthesis of stable and polydispersed AuNPs. Despite their antimicrobial and anticancer properties, AuNPs are well known for their excellent catalytic efficiency [106,107]. Similarly, the green biosynthesis of silver nanoparticles (AgNPs) was performed using various algae such as *Cystophora moniliformis*, *Caulerpa racemosa*, *Ecklonia cava*, and *Euglena gracilis*. Biosynthesized AgNPs were investigated for their antimicrobial, antioxidant and anticancer activity [108,109,110,111]. More examples of various algae-associated biosynthesized nanomaterials are listed in Table 1.

## 4. Nanomedical Applications of Seaweed Polysaccharides

The nanomedical applications of algal polysaccharides include drug delivery vehicles, tissue engineering scaffolds, antimicrobial agents, imaging agents, multiple theranostic agents, etc. Agar nanospheres were designed for the sustained delivery of bupropion HCl with an antidepressant drug (hydroxypropyl beta-cyclodextrin) as a permeability enhancer. Agar nanospheres were encapsulated with bupropion HCl via ionic gelation, and then drug encapsulation and release profiles were analyzed. For the in vivo studies in rats, the dug nanospheres were introduced through intratracheal spraying, which were later compared with the same dose of the drug delivered through intravenous and pulmonary delivery. The drug nanospheres had particle size of 320 ± 90 nm with a zeta potential of 29.6 mV, a polydispersity index of 0.85, a drug loading efficiency of 43% and a release efficiency of 66%. Compared to the normal drug solution, the bioavailability of the drug nanospheres improved from 0.25% to 86.69%. Based on the results, the bupropion-loaded agar nanospheres functioned as a biocompatible delivery system with systemic exposure and enhanced bioavailability [149].

Biopolymer-modified superparamagnetic have significant biomedical applications due to their synergistic effects including biocompatibility and fast responses to electromagnetic signals. Agar obtained from the red marine algae *Gelidium robustum* is utilized in the development of magnetite (Fe_3_O_4_) and cobalt ferrite (CoFe_2_O_4_) nanoparticles using the co-precipitation method at five different concentrations (1–5%). The agar-coated superparamagnetic nanoparticles with ≥ 3% agar concentration showed high saturation magnetization and excellent biocompatibility, making them promising candidates for bioimaging applications [150]. Another agar-based nanocomposite was made using Fe_3_O_4_ with a coprecipitation method, where agar was used as a polymer additive, which could be used in magnetic bioimaging [151].

Agar is used to form a bioactive agar copper sulfide (CuS) nanocomposite film by dispersing CuS nanoparticles (CuS NP) in the agar matrix. The addition of CuS NP decreased the swelling ratio and moisture content of the composite films; however, an increase in the water solubility, mechanical strength and water vapor barrier properties are observed. The in vitro analysis on skin fibroblast L929 cell lines showed the excellent biocompatibility of CuS NP and nanocomposite films with cell viability above 90%. Moreover, when tested against *E. coli*, *L. monocytogenes* and food-borne pathogenic bacteria, they exhibited characteristic antibacterial activity, which showed the potential of this nanocomposite in food packaging and biomedical applications [152]. Another agar-based biocompatible composite material was prepared in combination with magnetic iron oxide nanoparticles, graphite and sodium aluminum in different proportions. The prepared agar-nanocomposite-based composites displayed remarkable antimicrobial activities against *Escherichia coli*, *Klebsiella pneumoniae* and *Staphylococcus aureus* [151].

Another interesting investigation was conducted into the development of artificial vitreous humor. Herein, hyaluronic acid (HA)-agar-based hydrogels are compared with bovine vitreous humor, in terms of their rheological and drug or nanoparticle migration properties. Based on three polymer loads (high, medium, and low) gel compositions were made (VH, VM, and VL); then, their characteristics were evaluated. The viscoelastic properties were analyzed using oscillatory rheology, and the migration of nanoparticles through the gels was evaluated using multiple particle tracking. Based on the results, comparable rheological behavior was observed between the hydrogel and bovine vitreous. Additionally, tracking evaluations, optimal size–charge analysis and a model drug mobility test with a custom-built eye model were performed to evaluate the potential of the hydrogel. The low-viscosity HA-agar gels mimic both the rheological and adhesive properties of the vitreous humor and also supports the nanoparticle and drug migration through biological vitreous humor. For the in vitro evaluation, fluorescein dye was injected into the vitreous chamber of the model eye and, based on the permeation, the clearance percentage was measured after 24 h by quantifying the amount entered in the anterior chamber. Considering the fast degeneration of bovine ocular tissue, this artificial hydrogel offers long-term use in ex vivo models to study particle migration and clearance, while working towards a suitable alternative long-term evaluation to in vivo intravitreal pharmacokinetic studies [153].

Wound healing applications using agar hydrogels represent another promising area where silver nanoparticles are excellent antimicrobial agents. A wound-healing membrane hydrogel was developed using silver ions (Ag^+^) dispersed in polymer matrix made of agar, carboxymethyl cellulose (CMC), polyethylene glycol (PEG) and polyvinylpyrrolidone (PVP). The hydrogel had a high degree of swelling and excellent cytocompatibility, and the membranes were tested in vivo rabbit models, which displayed improved wound-healing properties [154].

Funoran and porphyran are structurally similar polysaccharides. Porphyran is utilized as a reducing agent and capping agent for the synthesis of gold nanoparticles. These porphyran coated nanoparticles were tested on the human glioma cell line (LN-229) as compared to native porphyran, where the nanoparticle showed enhanced cytotoxicity. When evaluated for their drug carrying capacity as a DOX carrier and compared with DOX drug itself, DOX-loaded porphyrin-coated nanoparticles exhibited improved cytotoxicity for LN-229 cells [155].

In a similar study, ovalbumin, along with porphyrin, is used as an encapsulating material for the delivery of resveratrol. These nanoformulations showed enhanced stability of dispersion, RES protection and excellent intestinal release. Furthermore, they also showed excellent inhibiting activity toward HeLa and HepG2 cell lines [156]. Apart from drug delivery, porphyran nanoparticles were successfully used as a non-viral gene delivery vehicle. Porphyran nanopolysaccharides, which are cationically modified with polyethyleneimine, are used for the delivery of plasmid to human umbilical cord mesenchymal stem cells, exhibiting high biocompatibility and excellent transfection efficiency [157].

Similarly, porphyrin-polysaccharide-derived carbon dots were used for the co-delivery of gene combinations for neuronal induction from ectodermal mesenchymal stem cells. Seven combinations of genes were used in this investigation, and high transfection efficiency was observed, which is comparable to that of commercially available transfection agents such as PEI (25 kDa) and Lipofectamine2000 [158].

Carrageenans are well known for their biocompatibility, hydrophilicity, cellular adhesion and proliferation properties. Similarly, hydroxyapatite also showed biocompatibility, bioactivity and bone regeneration, along with osteoconductivity. Thus, the combination of carrageenans with hydroxyapatite created an excellent biomaterial for bone tissue engineering with synergistic properties from both components. In vitro investigations of human osteoblasts showed cytocompatibility, cellular attachment, proliferation possibility and cell-spreading capacity. This novel nanomaterial could be used as an injectable bone substitute for bone regeneration [159]. Carrageenans can be degraded into lower-molecular-weight oligosaccharides using enzymes called carrageenans, with various biological and physiological activities including anti-tumor, anti-inflammation, anti-viral, anti-coagulation effects, etc. [160]. An injectable bone substitute was designed with carrageenan and nano-hydroxyapatite for bone regeneration purposes. The nanocomposite was prepared via chemical cross-linking, and the in vitro evaluation was conducted on mammalian cells and bacteria to analyze the properties. Various formulations containing 1%, 1.5%, and 2.5% carrageenan and 60% nano-hydroxyapatite were then tested to analyze human bone regeneration and antimicrobial functions. The MTS viability test of nanocomposites on osteoblasts resulted in cell adhesion and viability for various formulation types. This composite acted as an innovative biomaterial for enhanced osteoblast responses with excellent antibacterial activity [161].

**Figure 5:**
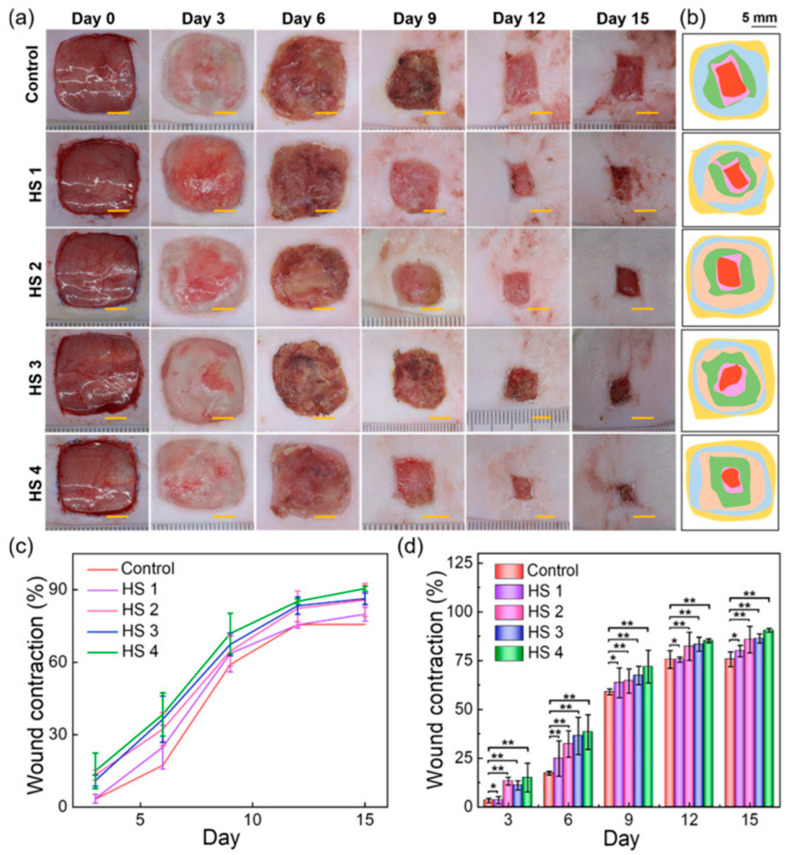
Wound healing process (**a**) Photographs illustrating the progression of wound healing in the control and treatment groups (HS 1–HS 4) over the 15-day observation period. (**b**) Corresponding wound area tracing and simulated wound bed closure at days 0, 3, 6, 9, 12, and 15 post-treatment. Scale bar: 5 mm. (**c**,**d**) Quantitative analysis of wound contraction for the control and experimental groups at the indicated time points. Data are expressed as mean ± standard deviation (n = 5). Statistical significance is indicated as * *p* < 0.05 and ** *p* < 0.01. Reproduced from Ref. [166] under the Creative Commons CC BY 4.0 license.

Carrageenan is also used in nanodrug delivery applications of curcumin as an inflammation inducer in animal models to cure inflammatory disorders [162]. A study on the fabrication of carrageenan incorporated nanotubes was conducted by Chough et al. The nanotubes were made using α-lactalbumin, bovine serum albumin and chitosan, with κ-carrageenan. The potential of these nanotubes as a drug carrier was tested according to their encapsulation and delivery of curcumin. These carrageenan nanotubes showed encapsulation efficiencies of about 40–45% and successive releasing capacity in physiological conditions up to 300 μg/mL, with good biocompatibility [163]. A carrageenan hydrogel was developed by incorporating AgNPs. The in situ synthesis of AgNPs changed the swelling properties, viscosity and gelling characteristics. Due to the release of silver nanoparticles, the hydrogel exhibited antimicrobial properties, which are recommended for wound-healing applications [164]. A wound-healing hydrogel (Carr/GSE/SNP) was developed by utilizing κ-carrageenan with chitosan-capped sulfur nanoparticles and grapefruit seed extract. The hydrogel film displayed antibacterial activity and showed high biocompatibility against L929 mouse fibroblast cell lines. The in vivo wound healing analysis via hematoxylin and eosin staining on Sprague–Dawley rats by carrageenan-based hydrogel films compared to control (povidone-iodine—Tegaderm) displayed an excellent wound healing effect of 1.3% wound area after two weeks. Moreover, the histological examination presented the appearance of the completely healed epidermis, proving the successful wound-healing capacity of the hydrogel [165]. Another novel 3D-printed hydrogel scaffold was prepared by incorporating flavanone (FLA)-loaded ZIF-8 nanoparticles (FLA@ZIF-8 NPs) with a composite of k-carrageenan (KC) and konjac glucomannan (KGM). This scaffold forms a mechanically stable dual network structure through the chelation of KC with potassium ions and intermolecular hydrogen bonding, providing structural integrity and wound environment adaptation. The integration of the pH-responsive FLA@ZIF-8 nanosystem enables controlled decomposition in acidic conditions, facilitating the sustained release of FLA and Zn 2^+^ ions and ensuring antimicrobial activity. The 3D-printed structure ensures the precise special distribution of the nanoparticles in the scaffold, enhancing drug loading capacity and release control. The in vitro evaluations of Sprague–Dawley rats displayed good cytocompatibility. As shown in Figure 5, the in vivo wound healing experiments confirmed the accelerated wound closure and tissue regeneration compared with control treatments, demonstrating the wound management potential of the carrageenan-based nanocomposite [166].

Kappa-Carrageenan (κ-carrageenan) and silver nanoparticles were used for the synthesis of bio–nano composite hydrogel beads. Compared to the pure carrageenan, this bio-nano composite hydrogel showed less swelling. Due to the presence and release of silver ions, this nanocomposite showed good antibacterial activities against *Staphylococcus aureus*, Methicilin Resistant *Staphylococcus aurous*, *Pseudomonas aeruginosa* and *Escherichia coli*. Moreover, the cytotoxicity of bio–nano composite hydrogels displayed a non-toxic effect of concentrations up to 1000 μg/mL, making it suitable for biomedical applications [167]. Another injectable hydrogel scaffold was created by combining κ-carrageenan, chitosan, and poly (NIPAM) with gold nanoparticles. Along with the cytocompatibility, cell growth and attachment properties, the presence of gold nanoparticles created a conducive environment inside the hydrogels, supporting the growth of the cells, which is reinforced by the in vitro investigation of MG-63 cells [168].

An electroconductive material was made utilizing carrageenan in combination with starch and poly-(3,4-ethylenedioxythiophene), enabling the direct delivery of electrical, electrochemical and electromechanical signals to biological tissues. This nanocomposite displayed excellent biocompatibility and tunable electrical conductivity. Here, κ-carrageenan acted as a doping agent to enhance the biocompatibility and the electrical response of the conductive polymer; additionally, it enhanced the compressive strength. The synergistic effects of electrical properties and biocompatibility enable the use of this nanocomposite in biomedical applications [169].

Alginate is extensively used in the pharmaceutical industry due to its biocompatibility, biodegradability and carrier capacity (drug or nanoparticles). It is also utilized for the immobilization of specific molecules such as enzymes or peptides [170]. A sodium-alginate-based hydrogel was developed by incorporating polyacrylamide, polyacrylic acid and different wt% of cloisite-30B modified clay with silver nanoparticles for the targeted delivery of paclitaxel. The hydrogel had an optimum encapsulation efficiency of about 72.66 ± 5.92%. The in vitro drug release and anticancer studies showed the potential of this hydrogel in cancer therapy [171]. Another investigation focused on edible agar films with nano–bacterial cellulose (BC). Various concentrations of agar (0, 3, 5, 8 and 10%, wt%) were used in the film preparation. The addition of the BC enhanced the crystallinity, tensile strength and thermal stability of the edible films. However, BC introduction significantly decreased moisture content, water solubility and water vapor permeability. These agar–BC edible films are perfect for food packaging [172]. An alginate-based magnetic CoFe_2_O_4_-nanoparticle-incorporated hydrogel bead was developed for drug delivery applications. The hydrogel beads were prepared via the coprecipitation method using caffeine as a new eco-friendly material to alkalinize the medium. The in vitro release of the beads on the MTT assay on U87 cell lines showed the biocompatibility of the alginate magnetic hydrogel beads [173].

For the retroviral drug delivery of the antiviral drug zidovudine, a system was developed using Pluronic F-68 (PF-68)-coated alginate conjugate nanoparticles. The developed nanosystem showed a loading efficacy of 29.5 ± 3.2% and the slow and sustained release of the zidovudine. The nano-drug delivery system showed excellent biocompatibility and impressive in vitro internalization efficiency [174]. Another drug delivery system in a nanoflower hierarchy was developed using cationic-β-CD as the core, along with alginate and chitosan as petals, for the delivery of 5-Fluorouracil (5-FU) with a drug-loading efficiency of up to 77.3%. From the in vitro release analysis on L929 cells, the nanoflowers showed controlled and sustained release of the drug and inhibition towards growth based on the drug concentration [175]. For the delivery of the anticancer drug doxorubicin (DOX), a nanodrug delivery system is developed using sodium alginate along with polyvinyl alcohol and bovine-serum-albumin-coated Fe_3_O_4_ nanoparticles. When the cytotoxicity of the nanosystem was tested using the MTT assay, it was found to be toxic to HepG2 cell lines and non-toxic to L02 cell lines. The designed system also showed faster drug release in an acidic environment, and most cancer cells show an acidic environment, making it a suitable smart-drug-delivery system [176].

Modified nonionic alginate polymers were also used for functionalizing various up-conversion nanoparticles (UCNPs) to create more stability, biocompatibility, and improved luminescence intensity for NIR imaging. Moreover, these nanosystems are capable of drug encapsulation. DOX-incorporated alginate-functionalized UCNPs were used simultaneously for drug release in KB cells and NIR imaging as a perfect theranostic system for future medical advances [177]. The combination of alginate, chitosan and folate hybrid system for the paclitaxel (PTX) delivery was another interesting idea. Various combinations of alginate–chitosan and folate–chitosan combinations were evaluated for their in vitro anticancer potential in HepG2 cells [178]. For the delivery of the anti-HIV drug zidovudine, another nanosystem was designed using hybrid nanoparticles of alginate and stearic acid–polyethylene glycol (SA-PEG). The various characterizations displayed their dendritic morphology (e.g., core and shell), a good encapsulation efficiency of 83.18 ± 1.22%, and a surface potential of −42.53 mV. Hemolysis and aggregation studies revealed that there was no lysis or aggregation in WBC, RBC and platelets. From the in vitro evaluations of Glioma, Neuro2a and Hela cells, the cytocompatible nature of the nanoparticles is observed. Moreover, the blood hemolysis and aggregation studies showed no cell lysis and aggregation in blood cells, which supports the use of this nanosystem as a good carrier for zidovudine delivery [179].

Fucoidan nanostructures, fucoidan coatings and fucoidan combinations with other biomaterials are widely explored in terms of surface functionalization, drug delivery and tissue engineering applications [180,181]. A nano scaffold for bone tissue engineering was fabricated using Genipin-crosslinked nanohydroxyapatite and hydroxypropyl chitosan in combination with fucoidan. The scaffold showed enhanced water solubility, excellent biocompatibility, and improved antioxidant and antibacterial activities. The introduction of fucoidan improved the osteoconductive and osteogenic properties of the nanocomposite scaffolds via electrostatic interactions. The adsorption of fucoidan on the scaffolds improved ALP activity in 7F2 osteoblast cells and promoted their mineralization, showing promising results in bone tissue engineering [182]. Another 3D bone tissue engineering scaffold was prepared by combining chitosan, natural nano-hydroxyapatite and fucoidan. The addition of nano-hydroxyapatite into the chitosan–fucoidan composite scaffold exhibited a suitable microarchitecture for cell growth and nutrient supplementation in mesenchymal stem cells (PMSCs). The use of a scaffold displayed good biocompatibility and excellent mineralization, making it suitable for bone regeneration [183]. Fucoidan is an inherent anticancer molecule, and it could be also used as a nanocarrier, coating material and targeting ligand with no systemic toxicity. These properties make it an excellent drug carrier for targeted drug delivery considering its P-selectin specificity [85].

**Figure 6:**
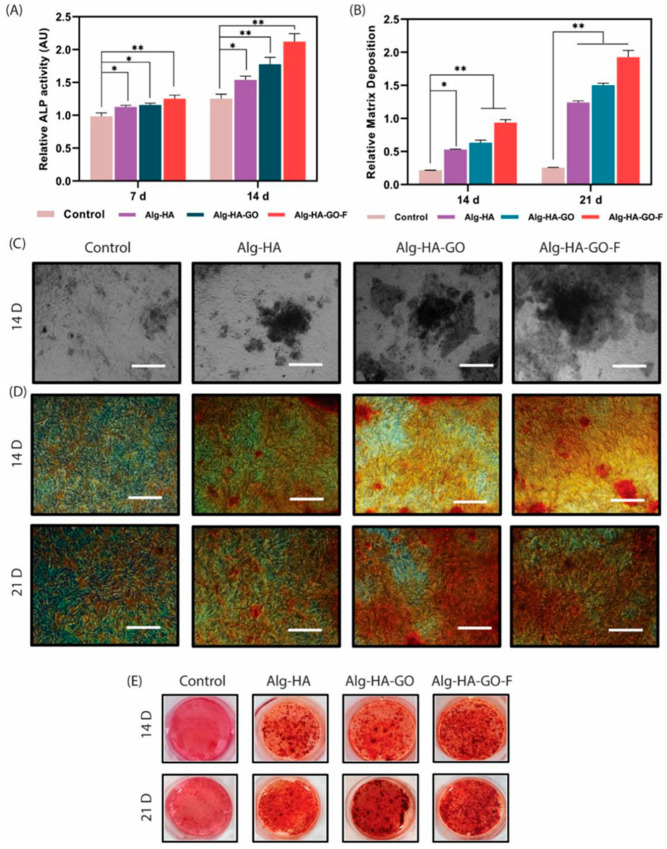
Osteogenic response of mesenchymal stem cells cultured on alginate-based composite scaffolds. (**A**) Alkaline phosphatase (ALP) activity measured after 7 and 14 days of culture (n = 3) for cells treated with Alg–HA, Alg–HA–GO, and Alg–HA–GO–F scaffolds. ALP activity was quantified via the detection of p-nitrophenol. (**B**) Quantitative mineralization assessment based on optical density measured at 570 nm following solubilization with cetylpyridinium chloride (scale bar: 150 μm). (**C**) Von Kossa staining of murine mesenchymal stem cells after 14 days of culture, demonstrating calcium deposition (scale bar: 150 μm). (**D**,**E**) Alizarin Red S staining of murine mesenchymal stem cells cultured for 14 and 21 days, illustrating progressive extracellular matrix mineralization. All data represent the mean ± standard deviation of three independent experiments, with * *p* < 0.05 and ** *p* < 0.01 relative to control cells. Reproduced from Ref. [185] under the Creative Commons CC BY 4.0 license.

A hydroxyapatite-fucoidan (HA-Fucoidan) nanocomposite system was developed as a bone tissue engineering scaffold, considering the bone repair and replacement properties of hydroxyapatite and the biocompatibility of fucoidan. The in vitro investigations of adipose-derived stem cells displayed a mineralization effect and enhanced gene expression of osteocalcin, osteopontin, collagen and runx-2. In vivo investigations in rabbit models showed bone regeneration in terms of osteoblast formation, which is an excellent bone scaffold characteristic [184]. Another bone graft substitute was prepared using Fucoidan from *Sargassum ilicifolium*. The novel multifunctional osteoinductive composite is prepared by combining fucoidan with calcium-crosslinked sodium alginate, nano-hydroxyapatite, and nano-graphene oxide (Alg–HA–GO–F). The in vitro evaluation using mesenchymal stem cells, as shown in Figure 6, demonstrated enhanced osteogenic differentiation, as evidenced by increased alkaline phosphatase activity and the upregulated expression of key osteogenic markers. These findings highlight the capacity of fucoidan to synergistically enhance the osteoconductive and osteoinductive properties of composite scaffolds, supporting its potential application in bone tissue engineering and regenerative medicine [185].

In the case of dextran-coated superparamagnetic iron oxide nanoparticles, fucoidan acted as an agent to extend the circulatory time of the magnetic MRI contrast agents. Being a biocompatible seaweed polysaccharide, fucoidan was efficient in inhibiting RES uptake and improved the circulatory half-life, which increased tumor uptake chances. When tested in subcutaneous (GL261) tumor mice models, the fucoidan with 89Zr-ferucarbotran in the superparamagnetic iron oxide nanoparticles showed excellent MRI signals, acting as promising MRI contrast agents and enabling imaging and magnetic hyperthermia [186].

A fucoidan-coated core–shell magnetic mesoporous silica drug carrier is utilized in the delivery of DOX towards cancer cells. Fucoidan coating of silica nanoparticles was achieved via metal–ligand complex coordination and fucoidan acted as a smart system for the pH-responsive delivery and magnetic-ion-oxide-based hyperthermia application. The nanosystem showed good drug loading and retention capability, along with smart delivery in the acidic environment and elevated temperatures (45 °C). The MTT and intracellular uptake studies of MDA-MB-231 cells proved the biocompatibility and internalization ability of the nanosystem. Additionally, the hyperthermia potential in the magnetic field enhances the therapeutic potential of this biocompatible smart drug delivery nanosystem [187]. A multi-stimuli-responsive nanosystem was designed using a combination of fucoidan with a cationic polypeptide–protamine nanoparticle for the delivery of DOX. In addition to fucoidans’ anticancer properties, this system had features including selective targeting by linking P-selectin, charge conversion, and stimuli-responsive properties. Additionally, the DOX release was triggered by enzymatic digestion and acidic intracellular pH. When tested against a metastatic breast cancer cell line (MDA-MB-231), this DOX-loaded nanosystem showed significant inhibitory effects [188]. A nanotheranostic system of DOX delivery along with phototherapy was developed by Kim et al. The combination of DOX-loaded fucoidan and AuNPS demonstrate the possibility of dual cancer therapy with this nanosystem. This stable nanosystem showed promising results on eye tumors both in vitro and in vivo and acted as a perfect nanotheranostic material [189]. Another multifunctional drug delivery system was developed by combining fucoidan and gold nanoparticles, where fucoidan acted as a capping and reducing agent. DOX-loaded fucoidan-capped AuNPs were utilized for drug delivery and photoacoustic imaging. The acidic environment triggered the release of DOX and on-time photoimaging using AuNPs and the theranostic potential of the nanosystem [190]. A layer-by-layer core–shell nanoparticle drug carrier system was achieved via the combination of poly(lactide-co-glycolide), poly-L-ornithine and fucoidan. The anticancer drug DOX was loaded into the poly(lactide-co-glycolide) core with good drug loading efficiency. The in vitro studies on the MCF-7 cell line showed the successful internalization of the carrier and controlled release of DOX [191]. In another study, a fucoidan–chitosan nanocarrier was designed for the topical delivery of methotrexate towards the treatment of skin-related inflammatory diseases. From the skin permeation studies, the nanosystem showed an anti-inflammatory effect and an increase in skin permeation, which enhanced the topical delivery of methotrexate [192]. Another fucoidan-based nanosystem was developed for immunomodulator-focused tumor therapy. A fucoidan–dextran-based iron oxide magnetic nanosystem was conjugated with an inhibitor (anti-PD-L1) and T-cell activators (anti-CD3 and anti-CD28) to identify and repair the immunosuppressive tumor microenvironment by giving a boost to tumor-infiltrating lymphocytes while targeting the magnetic navigation to the tumors [193].

Pro-oxidant therapy is a method of utilizing pro-oxidant drugs to trigger cytotoxic oxidative stress in cancer cells for cancer-specific treatment. Fucoidan and chitosan-based nanoparticles were used in combination with a pro-oxidant drug: piperlongumine was investigated to determine the oxidative stress in cancer cells. This nanosystem was developed for the intracellular delivery of the hydrophobic drug piperlongumine, which improved the water solubility and bioavailability of the drug. When tested in human prostate cancer cells, this nanosystem showed specific therapeutic action towards the cancer cells via induced reactive oxygen species generation [194]. Another investigation was conducted to explore the real-time tracking of autophagy; a bioactive fucoidan-coated plasmonic gold nanoparticle complex was developed and explored for cellular imaging on human oral squamous carcinoma cells (HSC3) via plasmonically enhanced Raman spectroscopy. This system enabled the tracing of the entire autophagic process from cell introduction to autophagic apoptosis. This nanosystem shows that the combination of nanocomposites and spectroscopic techniques can be used to explore abnormalities at the molecular level [195]. Although it does not originate from seaweed sources, the marine polysaccharide chitosan has also taken on a major role in the nanomedical field, in terms of drug delivery, wound healing, bioimaging and tissue engineering [196,197,198,199,200,201,202].

## 5. Recent Advances and Future Directions in Marine Polysaccharide Nanomedicine

The field of marine polysaccharide nanomedicine has seen extraordinary advancements in recent years, with several transformative studies showing the clinical potential of algal-derived nanomaterials. Recent studies highlight the potential of fucoidan–chitosan nanoparticles as efficient CRISPR-Cas9 delivery vehicles. These targeted systems demonstrate significantly enhanced gene-editing capabilities in hepatocellular carcinoma models through P-selectin binding, while showing markedly reduced off-target effects compared to conventional lipofectamine-based approaches [203,204]. In addition, Hwang et al. (2024) described 3D-printed electroconductive cardiac patches made from alginate-porphyran bioinks embedded with gold nanorods, which are capable of restoring myocardial function in infarcted rat models [205]. Another promising direction included sulfated ulvan nanoparticles from *Ulva Lactuca*, which demonstrated the neutralization of SARS-CoV-2 virions by mimicking ACE2 receptors, offering promising broad-spectrum antiviral protection [206,207]. Artificial intelligence has further revolutionized the field, with studies reporting machine learning models that predict carrageenan–doxorubicin binding affinities with high accuracy, enabling the accelerated design of personalized oncology nanocarriers [208]. In another recent investigation, self-healing agarose–cerium oxide nanocomposites showed autonomous wound closure in diabetic mice through combined ROS-scavenging and real-time pH-observing capabilities [209]. Complementing these therapeutic advances, a 2023 study also marked progress in sustainable production, with integrated biorefineries achieving the lower-waste extraction of fucoidan (up to ~94% yield) alongside concurrent biofuel production from brown seaweed biomass such as *Sargassum* sp. [210,211]. Despite these advances, challenges remain in clinical translation due to batch variability in algal polysaccharides [212] and the need for good manufacturing practices (GMP)-compliant automated synthesis platforms [213]. With the global algae-based drug market projected to grow at nearly 18 to 20% compound annual growth rate (CAGR) through 2030 [214,215], marine polysaccharides are poised to transform sustainable nanomedicine through continued innovations in precision targeting, AI-driven design, and circular bioeconomy integration. The improvement of marine-polysaccharide-based nanomedicine relies on a multidisciplinary combination of experimental procedure, but clinical translation is hindered by limited in vivo studies. Extensive evaluations of safety, efficacy, and immunological responses are urgently needed.

## 6. Conclusions

Macroalgae-derived polysaccharides have been intensively utilized in biomedical and nanomedical applications due to their structural diversity, biocompatibility, and functional versatility. The selection of algal polysaccharides for specific biomedical application is determined by their structure–property–function relationship. Non-sulfated polysaccharides are preferred in drug delivery, tissue engineering, wound healing, and cell encapsulation due to their biocompatibility, mechanical stability, and minimal intrinsic bioactivity, which allows for controlled therapeutic performance. In contrast, sulfated polysaccharides such as fucoidan and carrageenan are increasingly selected for bioactivity-driven applications including antimicrobial coatings, antiviral formulations, anticoagulant systems and anticancer nanotherapeutics, due to their higher sulfate content and polyanionic charge, enabling interactions with cellular membranes, proteins and pathogens. Agar and agarose play vital roles in diagnostic, separation, and controlled-release platforms due to their reversibility and structural uniformity. The integration of algal polysaccharides into nanosystems enhances their potential to contribute to enhanced biocompatibility and prolonged circulation times, along with producing antimicrobial, antiviral, anti-inflammatory, and anticancer effects. The synergistic interactions of polysaccharide-based nanomaterials enable theranostic opportunities, facilitating the real-time monitoring of therapeutic outcomes. While multiple studies display the materials’ promising in vitro and in vivo performance, detailed clinical validation is necessary to support the translation and commercialization of these nanoproducts. Ultimately, macroalgal polysaccharides represent sustainable and multifunctional biomaterials with strong potential to address current challenges in drug delivery, regenerative medicine, nanomedicine and advanced therapeutic design.

## Figures and Tables

**Table 1:** Algae-assisted green synthesis of nanomaterials and applications.

Algae	Nanomaterial	Size	Application. Target Pathogen/Catalytic Action	Reference
*Acanthophora* *specifera*	silver	33–81 nm	antimicrobial activity. *Staphylococcus aureus*, *Bacillus subtillis*, *Salmonella* spp., *Escherichia coli*	[112]
*Amphiroa* *fragilissima*	silver	-	antibacterial activity, *Escherichia coli*, *Bacillus subtilis*, *Klebsiella pneumonia*, *Staphylococcus aureus*, *Pseudomonas aeruginosa*	[113]
*Anabaena cylindrica*	copper oxide	3.6 nm	drinking water disinfection. *Escherichia coli*.	[114]
*Caulerpa racemosa*	silver	25 nm	catalytic activity towards methylene blue.	[115]
*Caulerpa serrulata*	silver	10 ± 2 nm	catalytic and antibacterial activity. *Staphylococcus aureus*, *Pseudomonas aeruginosa*, *Shigella* sp., *Salmonella typhi*, *Escherichia coli*	[116]
*Chlamydomonas reinhardtii*	cadmium sulphide	5 nm	photocatalysis towards organic dye degradation.	[117]
*Chlorella vulgaris*	silver	40–90 nm	synthesis of substituted quinolines.	[118]
*Chlorella vulgaris*	palladium	70 nm	catalytic activity for the synthesis of N-aryl piperazines.	[119]
*Cladophora vagabunda*	fluorescent carbon	42.78 nm	fluorescent material for biological activities. *Staphylococcus aureus*, *Escherichia coli*	[120]
*Galaxaura elongate*	gold	-	agriculture application- protection against plant pathogens.	[121]
*Galaxaura elongata*	gold	3.85–77.13 nm	antimicrobial applications. *Escherichia coli*, *Klebsiella pneumoniae*, MRSA, *Staphylococcus aureus*, *Pseudomonas aeruginosa*.	[122]
*Gelidium amansii*	silver	20–95 nm	antibacterial agents. *Staphylococcus aureus*, *Bacillus pumilus*, *Escherichia coli*, *Pseudomonas aeruginosa*, *Vibrio parahaemolyticus*, *Aeromonas hydrophila*	[123]
*Gelidium corneum*	silver	20–50 nm	antibiofilm, antimicrobial activity. *Candida albicans*, *Escherichia coli*	[124]
*Gelidiella acerosa*	gold	5.81–117.59 nm	antioxidant activity and antibacterial activity. *Escherichia coli*, *Serratia marcescens*, *Klebsiella pneumonia*, *Bacillus subtilis*	[125]
*Gracilaria birdiae*	silver	20.2–94.9 nm	antibacterial activity. *Escherichia coli*, *Staphylococcus aureus*	[126]
*Gracilaria crassa*	silver	122.7 nm	antibacterial activity. *Escherichia coli*, *Proteus mirabilis*, *Bacillus subtilis*, *Pseudomonas aeruginosa*.	[127]
*Gracilaria verrucosa*	gold	20–80 nm	anticancer activity. human embryonic kidney HEK-293 cell lines.	[128]
*Halymenia dilatata*	gold	16 nm	antioxidant, anticancer and antibacterial activity. *Aeromonas hydrophila*, human colorectal adenocarcinoma HT-29 cell line	[129]
*Laurencia* *catarinensis*	silver	39.41–77.71 nm	green chemistry.	[130]
*Neochloris* *oleoabundans*	silver	16.63 nm	antibacterial activity *Escherichia coli*	[131]
*Padina pavonia*	silver	49.58–86.37 nm	green synthesis.	[132]
*Polysiphonia* sp.	silver	5–25 nm	anticancer activity breast cancer MCF-7 cell line	[133]
*Portieria* *hornemannii*	silver	70–75 nm	anti fish pathogen activity *Vibrio harveyii*, *Vibrio parahaemolyticus*, *Vibrio alginolyticus*, *Vibrio anguillarum*	[134]
*Pseudopediastrum boryanum*	silver	-	antimicrobial activity *Proteus vulgaris*, *parapsilosis*, *Pseudomonas aeruginosa*, *Candida parapsilosis*, *Aeromonas hydrophila*, *Staphylococcus epidermidis*, *Candida parapsilosis*, *Candida albicans*	[135]
*Spyridia fusiformis*	silver	5–50 nm	antibacterial activity. *Escherichia coli*, *Klebsiella pneumonia*, *Staphylococcus aureus*, *Pseudomonas aeruginosa*	[136]
*Sargassum* *latifolium*	selenium zinc	22.31–95.16 nm	edible oils preservation via bio-reduction reaction to prevent oils oxidation and rancidity.	[137]
*Sargassum* *muticum*	zinc oxide	30–57 nm.	liver cancer therapy human liver cancer HepG2 cell line	[138]
*Sargassum* *polycystum*	copper oxide	-	antimicrobial and anticancer activities. *Pseudomonas aeruginosa*, *Aspergillus niger.* MCF-7 cells	[139]
*Spatoglossum* *asperum*	silver	28.8 nm	antifungal activity. *Aspergillus flavus*, *Candida albicans*, *Candida tropicalis*, *Trichophyton mentagrophytes*	[140]
*Spirulina platensis*	palladium	10–20 nm	adsorption activity in lead removal.	[141]
*Spyridia filamentosa*	silver	20–30 nm	antibacterial and anticancer activity. *Staphylococcus* sp. and *Klebsiella* sp. MCF-7 cells.	[142]
*Trichodesmium* *erythraeum*	silver	26.5 nm	antioxidant, drug-resistant antibacterial activity, and cytotoxicity activity. *Staphylococcus aureus*, *Proteus mirabilis*, *E. coli* (Amikacin^R^), *S. aureus* (Tetracycline^R^), *S. pneumoniae* (Penicillin^R^). HeLa, MCF-7 cell lines	[143]
*Ulva armoricana*	silver	215 nm	anticancer, antimicrobial activity. Balb/3T3 mouse embryo fibroblasts. *Escherichia coli*, *Pseudomonas aeruginosa*, *Staphylococcus aureus*, *Staphylococcus epidermidis*	[144]
*Ulva armoricana*	gold	~200 nm	catalytic activity towards the reduction of 4-nitrophenol.	[145]
*Ulva flexuosa*	iron oxide	12.3 ± 1.7 nm	antimicrobial activity. *Brachionus rotundiformis.*	[146]
*Ulva intestinalis*	gold silver	17.8± 2.7 nm 14.2± 2 nm	anticancer activity, therapeutic vaccines. Raw 264.7 cells, HL-60 cells, HL-60 cells	[147]
*Ulva lactuca*	gold silver	7.9 ± 1.7 nm 31 ± 8 nm	colorectal cancer therapy. colon cancer cell lines HT-29 and Caco-2	[148]

## Data Availability

No new data were created or analyzed in this study. Data sharing is not applicable to this article.
